# A reinforcement learning diffusion decision model for value-based decisions

**DOI:** 10.3758/s13423-018-1554-2

**Published:** 2019-03-28

**Authors:** Laura Fontanesi, Sebastian Gluth, Mikhail S. Spektor, Jörg Rieskamp

**Affiliations:** Faculty of Psychology, University of Basel, Missionsstrasse 62a, 4055 Basel, Switzerland

**Keywords:** Decision-making, Computational modeling, Bayesian inference and parameter estimation, Response time models

## Abstract

Psychological models of value-based decision-making describe how subjective values are formed and mapped to single choices. Recently, additional efforts have been made to describe the temporal dynamics of these processes by adopting sequential sampling models from the perceptual decision-making tradition, such as the diffusion decision model (DDM). These models, when applied to value-based decision-making, allow mapping of subjective values not only to choices but also to response times. However, very few attempts have been made to adapt these models to situations in which decisions are followed by rewards, thereby producing learning effects. In this study, we propose a new combined reinforcement learning diffusion decision model (RLDDM) and test it on a learning task in which pairs of options differ with respect to both value difference and overall value. We found that participants became more accurate and faster with learning, responded faster and more accurately when options had more dissimilar values, and decided faster when confronted with more attractive (i.e., overall more valuable) pairs of options. We demonstrate that the suggested RLDDM can accommodate these effects and does so better than previously proposed models. To gain a better understanding of the model dynamics, we also compare it to standard DDMs and reinforcement learning models. Our work is a step forward towards bridging the gap between two traditions of decision-making research.

Research on value-based decisions investigates how individuals value options and make decisions between them. Every-day decisions can be based on descriptive information, such as when choosing a restaurant based on reviews, or on personal experience, such as when choosing a restaurant based on previous visits. Reinforcement learning (RL; Sutton & Barto, 1998) describes the processes involved in the latter case, and specifically how the value associated with an option is updated following reward or punishment.

In the past decades, substantial progresses in understanding the mechanisms of RL have been made both in psychology (e.g., Bechara, Damasio, Damasio, & Anderson, 1994; Erev, 1998; Estes, 1950; Luce, 1959; Rieskamp & Otto, 2006; Yechiam & Busemeyer, 2005) and neuroscience (e.g., Dayan & Daw, 2008; Frank, Seeberger, & O’Reilly, 2004; Holroyd & Coles, 2002; Niv, 2009; Schultz, Dayan, & Montague, 1997). Within this framework, computational models can be used to infer latent value representations and psychological constructs (Lewandowsky & Simon, 2010), for instance, the reliance on more recent or past feedback (often referred to as the *learning rate*). RL models usually have two components: a learning component, that describes how past information is integrated with newly received feedback to update options’ subjective values, and a choice model, that maps the subjective values associated with the options to the final choice probabilities. Despite providing a good fit to choice data, this mapping function (e.g., the soft-max choice rule) does not provide a description of the cognitive processes that lead to a specific decision. Fortunately, these mechanisms can be revealed by simultaneously inspecting choices and response times (RTs). For example, making the same choice faster or slower can indicate less or more decision conflict, respectively (Frank, Samanta, Moustafa, & Sherman, 2007). Furthermore, choices and RTs might be differently affected under different conditions, and those cognitive processes that only affect RTs would be overlooked by models based on choices alone.

Sequential sampling models (SSMs; for an overview, see Bogacz, Brown, Moehlis, Holmes, & Cohen, 2006; Smith & Ratcliff, 2004) are process models that aim to describe the cognitive computations underlying decisions and allow predicting choices and RTs in a combined fashion. SSMs define decision-making as an integration-to-bound process: When deciding between two options, noisy evidence in favor of one over the other option is integrated over time, and a response is initiated as soon as the evidence reaches a pre-set threshold. Cautious decision-makers increase their threshold to make more accurate, but at the same time slower, decisions. On the other hand, if the situation requires to respond as quickly as possible, the threshold can be lowered at the cost of accuracy. When confronted with an easy decision (i.e., between a very good and a very bad option), the integration (or *drift*) rate is higher, leading to faster and more accurate decisions. SSMs have been successfully applied in many psychological domains (for an overview, see Ratcliff, Smith, Brown, & McKoon, 2016), including both perceptual and value-based decision-making (e.g., Busemeyer & Townsend, 1993; Usher & McClelland, 2001). In particular, the diffusion decision model (DDM; Ratcliff, 1978), the dominant model in perceptual decision-making, has gained particular popularity in value-based decision-making research (Summerfield & Tsetsos, 2012). Thus, the DDM has been used to directly compare perceptual and value-based choices (Dutilh & Rieskamp, 2016), and it has been extended to account for and to model eye-movement data in consumer-choice behavior (Krajbich Krajbich, Armel, & Rangel, 2010; Krajbich, Lu, Camerer, & Rangel, 2012). Moreover, building on the discovery of a neural correlate of the integration-to-bound process during perceptual decisions in non-human primates (Gold & Shadlen, 2001), SSMs have also been used to link behavioral and neural measures, such as the decision threshold to activity in the striatum (Forstmann et al., 2008; Gluth, Rieskamp, & Büchel, 2012; van Maanen, Fontanesi, Hawkins, & Forstmann, 2016).

While significant progress has been made in describing the processes underlying value-based decision-making, previous work mainly focused on situations in which rewards are not provided after each choice. SSMs typically assume that the subjective value associated with some option is stable in time and that repeated choices involving the same option do not affect its subjective valuation. The assumption of stable preferences might hold in many choice situations, but is presumably violated when some kind of feedback is received after every choice. In these cases, SSMs should be extended by adding a learning component.

To overcome the limitations of both SSMs (i.e., the absence of learning processes) and RL models (i.e., the absence of mechanistic decision processes), new models need to be developed. The goal of the present work is to propose a new computational cognitive model that describes both the processes underlying a single decision (by relying on the SSM approach of decision-making) and how these are influenced by the learning of subjective values of options over time (by relying on the RL framework). So far, only few attempts have been made to combine these two approaches (Frank et al., 2015; Pedersen, Frank, & Biele, 2017). In particular, these studies have proposed variants of the DDM in which an RL rule is used to update the subjective values, and these values in turn are mapped to trial-specific DDM parameters in a meaningful way (e.g., the difference in subjective values is mapped to the drift rate). Notably, in these studies, only the reward differences between options were manipulated, but not the mean values of different pairs of options. However, mean values have been reported to influence the speed of decisions (Palminteri, Khamassi, Joffily, & Coricelli, 2015; Pirrone, Azab, Hayden, Stafford, & Marshall, 2017; Polania, Krajbich, Grueschow, & Ruff, 2014), and could therefore be an important modulating factor of decisions during learning. Finally, an open question remains whether the subjective-value differences map linearly (as previously proposed) or non-linearly to the DDM parameters (more similarly to common decision rules in RL models, such as the soft-max choice rule).

In the present work, we propose a learning task in which not only value differences but also the mean values across different pairs of options are manipulated. We first test behavioral, cross-trial effects related to these manipulations, and develop a combined reinforcement learning diffusion decision model (RLDDM) that captures the observed learning and value-based behavioral effects. We then compare our model qualitatively and, whenever possible, quantitatively to other classes of models. We show that some of the value-based effects would have remained unnoticed if only choices but not RTs were taken into account—in particular those that are related to the mean value of pairs of options. Finally, we perform a rigorous model comparison analysis that illustrates the predictive advantages of the new model and provides insights into the cognitive processes underlying value-based decision-making during learning.

## Methods

### Participants and procedure

A total of 32 participants (24 female, age: 18–36, *M* = 22.36, *SD* = 2.14) completed the experiment. Participants were mainly psychology students recruited through the subject pool of the Faculty of Psychology of the University of Basel. Participation in the experiment was possible for partial fulfillment of course credits or cash (20 Swiss francs per hour). In addition, a monetary bonus corresponding to the performance in the experiment was awarded. Before starting the experiment, participants gave informed consent, as approved by the institutional review board of the Faculty of Psychology, University of Basel. The instructions of the task were presented directly on the screen. Information about participants’ gender, age, handedness, and field of study were also requested on-screen before starting the task. Since an accuracy above 56% across 240 trials is unlikely due to random behavior alone, according to a binomial test (*p* < 0.05), only participants who surpassed this threshold were included in the analyses. Raw data and scripts will be made available upon publication of the manuscript at https://osf.io/95d4p/.

### Learning paradigm

The paradigm was a multi-armed bandit problem (Sutton & Barto, 1998). A total of four options per block were presented and participants chose between two of them in each trial. Options were randomly assigned either to the left or to the right of a fixation cross, and could be chosen by pressing either Q (for left) or P (for right) on the keyboard. After each choice, participants saw both options’ rewards (i.e., full feedback) and collected the chosen option’s reward. At the end of the experiment, the accumulated reward, divided by 1400, was paid in Swiss francs to the participants as a bonus (e.g., if they collected 7000 points, they received 5 Swiss francs). On average, participants gained a bonus of 8.10 Swiss francs.

**Fig. 1:**
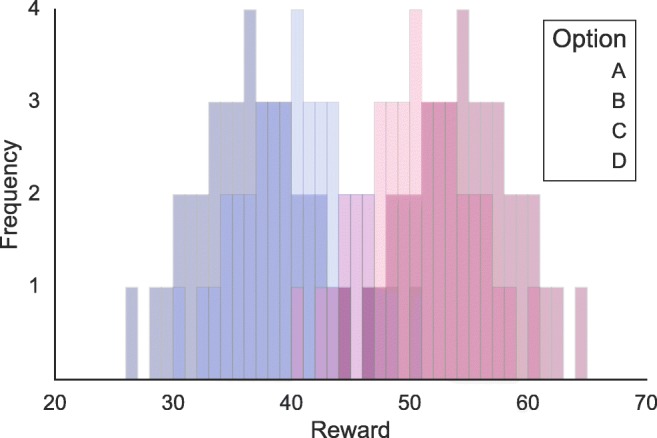
Reward distribution of the options **A**, **B**, **C**, and **D** in a learning block

Participants completed three experimental blocks of 80 trials, for a total of 240 trials. The payoffs of each option were not fixed but varied and were approximately normally distributed (Fig. 1). The mean rewards of the options in each block were 36, 40, 50, and 54 for options A, B, C, and D, respectively. The standard deviation was 5 for all options. The payoffs were rounded to the unit, and were controlled to have representative observations (i.e., each participant observed the same outcomes in a different order, and the sample mean of each option was equal to the generating mean). The order of the payoffs of a single option was different in each block, and options were associated with four new visual stimuli (see below for a description of the visual stimuli), so that the options had to be learned again in a new block.

**Fig. 2:**
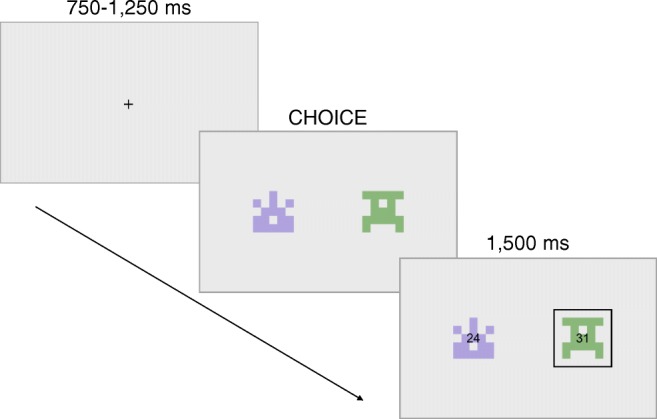
Example of a single trial: First, a fixation cross is shown from 750 to 1250 ms; then, two of the four options are shown and a choice has to be made; finally, the reward corresponding to both options is presented, and the reward corresponding to the chosen option (highlighted by a *black rectangle*) is collected

Each trial (Fig. 2) was separated by a fixation cross, presented for 750–1250 ms. The options were presented for up to 5000 ms. If a response was faster than 150 ms or slower than 3000 ms, the trial was discarded and a screen reminding to be slower or faster, respectively, was presented for 5000 ms after the participant’s response. Otherwise, the feedback was presented for 1500 ms.

### Design

In each learning block, only four of the six possible pairs of options were presented: AB, AC, BD, and CD (but not AD and BC). The order was pseudo-randomized so that the same pair would not be presented more than three times in a row. Presenting these four couples of options allowed us to test whether our model can predict two established behavioral effects of reward-based decision-making in addition to the learning effects. Previous studies have shown that, when deciding among options that have similar values (i.e., difficult choices), people tend to be slower and less accurate (e.g., Dutilh & Rieskamp, 2016; Oud et al., 2016; Polania et al., 2014). We will refer to this effect as the *difficulty* effect. In our study, difficulty, given by the mean value difference, was low in pairs AC and BD (the difference was 14 on average), and high in pairs AB and CD (the difference was 4 on average). Previous studies have also shown that absolute shifts in value can affect decision speed without necessarily changing accuracy (e.g., Palminteri et al., 2015; Pirrone et al., 2017; Polania et al., 2014): Participants tend to be faster when deciding between two higher-valued options as compared to two lower-valued options. We will refer to this effect as the *magnitude* effect. In our study, magnitude, given by the mean value of the pairs of options, was lowest in pair AB (38), followed by AC (43), BD (47), and CD (52). Finally, we refer to the *learning* effect as the improvement in performance throughout the trials. In this study, each pair was presented for 20 trials per block, and each option was presented in 40 trials per block (since each option is included in two different pairs).

### Stimuli

During the experiment, each participant saw a total of twelve different figures (four in each block) representing the options. The figures were matrices of 5×5 squares of which 17 were colored, arranged symmetrically along the vertical axis. To control for visual salience, we selected 12 evenly spaced colors in the HSL_UV_ space. A black rectangle was drawn around the chosen option at feedback presentation to highlight the collected reward. The experiment was programmed and presented using PsychoPy (Peirce, 2007).

### Cognitive models

In total, we estimated three classes of computational models: RL models, the DDM, and combinations of the two, RLDDM (some of which were previously proposed by Pedersen, Frank, and Biele (2017)). In the next sections, we present each class of models in detail.

#### Reinforcement learning models

RL models assume that the subjective values associated with the options are updated in each trial after experiencing a new reward (i.e., the reward feedback). These subjective values are then mapped to the probability of choosing one option over the other: Options with higher subjective values are chosen more often. Participants can differ in how much weight they give to new compared to old information: When more weight is given to old information, they are less affected by sudden changes in the rewards. They can also differ in how sensitive they are to subjective value differences: When they are very sensitive, their choices become more deterministic as they tend to always choose the option with the highest value. These two constructs, the stability of the subjective values and the deterministic nature of choices, are formalized in RL models by two parameters. The learning rate *η* (with 0 ≤ *η* ≤ 1), and the sensitivity *𝜃* (with *𝜃* ≥ 0). The learning rate is the weight that is given to new information when updating the subjective value. When *η* is close to 0, the old subjective value remains almost unchanged (implying that even observations dating far back are taken into account), whereas when *η* is close to 1, the new subjective value almost coincides with the new information (implying that earlier observations are heavily discounted). The sensitivity parameter regulates how deterministic the choices are. With a higher *𝜃*, choices are more sensitive to value differences, meaning that subjectively higher-valued options will be chosen over lower-valued options with higher probability.

On each trial, the subjective values *Q* of the presented options are updated following the so-called *delta learning rule*:

1 $$  Q_{t} = Q_{t-1} + \eta \cdot (f_{t} - Q_{t-1}) $$

where *t* is the trial number, and *f* is the experienced feedback. In the first learning block, Q-values were initialized at 27.5. This value was the average value shown in the task instructions at the beginning of the experiment, which was the same for all participants. In the subsequent learning blocks, the Q-values were initialized at the mean values learned in the previous blocks. We reasoned that adjusting initial Q-values according to prior knowledge is more realistic than simply initializing them at zero. Indeed, preliminary model estimations revealed that all models provided better fits when adjusting Q-values to prior knowledge. Choices in each trial are predicted by the soft-max choice rule:

2 $$  p_{t} = \frac{e^{\theta Q_{\text{cor}}}} {(e^{\theta Q_{\text{cor}}} + e^{\theta Q_{\text{inc}}})} $$

where *p* is the probability of choosing the option with the highest mean reward, and *Q*_cor_ and *Q*_inc_ are the subjective values of the options with a higher and lower mean reward, respectively.

Building on the simplest RL model, we took into account models that incorporate all possible combinations of two additional mechanisms, one concerning the learning rule and one concerning the choice rule. The first alternative mechanism allows *η* to differ depending on the sign of the reward prediction error. The reward prediction error is the difference between the feedback *f*_*t*_ and the previous reward expectation *Q*_*t*− 1_. Previous studies have found differences in learning rates for positive and negative reward prediction errors (Gershman, 2015) and have related this feature to optimism bias (Lefebvre, Lebreton, Meyniel, Bourgeois-Gironde, & Palminteri, 2017). The second mechanism allows *𝜃* to increase as a power function of how many times an option has been encountered before (as in Yechiam & Busemeyer, 2005) so that choices become more deterministic throughout a learning block:

3 $$  \theta_{t} = \left( \frac{n}{b}\right)^{c} $$

where *n* is the number of times an option has been presented, *b* (with *b* > 0) is a scaling parameter, and *c* (with *c* ≥ 0) is the consistency parameter. When *c* is close to 0, *𝜃* reduces to 1 and is fixed in time, while higher values of *c* lead to steeper increase of sensitivity throughout learning.

#### Diffusion decision model

The DDM assumes that, when making a decision between two options, noisy evidence in favor of one over the other option is integrated over time until a pre-set threshold is reached. This threshold indicates how much of this relative evidence is enough to initiate a response. Since the incoming evidence is noisy, the integrated evidence becomes more reliable as time passes. Therefore, higher thresholds lead to more accurate decisions. However, the cost of increasing the threshold is an increase of decision time. In addition, difficulty affects decisions: When confronted with an easier choice (e.g., between a very good and a very bad option), the integration process reaches the threshold faster, meaning that less time is needed to make a decision and that decisions are more accurate. The DDM also assumes that a portion of the RTs reflects processes that are unrelated to the decision time itself, such as motor processes, and that can differ across participants. Because of this dependency between noise in the information, accuracy, and speed of decisions, the DDM is able to simultaneously predict the probability of choosing one option over the other (i.e., accuracy) and the shape of the two RT distributions corresponding to the two choice options. Importantly, by fitting the standard DDM, we assume that repeated choices are independent of each other, and discard information about the order of the choices and the feedback after each choice. To formalize the described cognitive processes, the simple DDM (Ratcliff, 1978) has four core parameters: The drift rate *v*, which describes how fast the integration of evidence is, the threshold *a* (with *a* > 0), that is the amount of integrated evidence necessary to initiate a response, the starting-point bias, that is the evidence in favor of one option prior to evidence accumulation, and the non-decision time *T*_*er*_ (with 0 ≤ *T*_*er*_ < RT_min_), the part of the response time that is not strictly related to the decision process (RT = decision time + *T*_*er*_). Because, in our case, the position of the options was randomized to either the left or the right screen position, we assumed no starting-point bias and only considered drift rate, threshold, and non-decision time. Within a trial, evidence is accumulated according to the diffusion process, which is discretized in finite time steps according to:

4 $$  x_{i + 1} = x_{i} + \mathcal{N}(v \cdot dt, \sqrt{dt}), x_{0} = a/2 $$

where *i* is the iteration within a trial, and a response is initiated as soon as *x* ≥ *a* or *x* ≤ 0 (i.e., the evidence reaches the upper or the lower thresholds, respectively). The time unit *dt* is assumed to approach 0 in the limit (when *dt* = 0, the integration process is continuous in time). Choices are given by the value of *x* at the moment of the response (e.g., correct if *x* ≥ *a*, incorrect if *x* ≤ 0).

In total, we fit three versions of the DDM, varying in the number of free between-condition parameters. The first DDM had separate *v* s for difficult and easy choices, to allow accounting for the difficulty effect: Higher *v* s lead to faster and more accurate responses. The second model is as the first, but also has separate *a* s for option pairs with a higher or lower mean reward. This model variant allows accounting for the magnitude effect: Lower *a* s lead to faster, but not much more accurate decisions (Forstmann et al., 2011). This would explain the magnitude effect as a reduction of cautiousness: When confronted with more attractive options, individuals reduce their decision times (and therefore the time to the reward) by setting a lower threshold. The third model is as the second, but has also separate *v* s for option pairs with higher or lower mean reward, to check whether the magnitude effect is attributed only to a modulation of the threshold (i.e., cautiousness) or also to a modulation of the drift rate (i.e., individuals are better at discriminating two good options compared to two bad options).

#### Reinforcement learning diffusion decision models

The goal of our work is to propose a new model that overcomes the limitation of both the SSM and the RL frameworks. Therefore, we propose an RLDDM that is a combination of these two classes of models. The RLDDM simultaneously predicts choices and response times and describes how learning affects the decision process. Here, the DDM is tightly constrained by the assumed learning process: Instead of considering all choices as independent and interchangeable, the relationship between each choice, the experienced reward feedback, and the next choice is taken into account. The RLDDM assumes that, as in the RL framework, the subjective values associated with the options are updated after experiencing a reward feedback. The decision process itself is described by the DDM. In particular, the difference between the updated subjective values influences the speed of evidence integration in the next trial: When the difference is higher, as it might happen after experiencing several feedback, the integration becomes faster, leading to more accurate and faster responses. To formalize these concepts, we built a DDM in which the drift rate parameter is defined on each trial as the difference between the subjective values that are updated via the learning rule of RL models. The first and simplest RLDDM has four parameters (similarly to Model 1 in Pedersen et al.,, 2017): one learning rate *η* to update the subjective values following Eq. 1, a scaling parameter *v*_mod_ to scale the difference between values, one threshold *a*, and one non-decision time *T*_*er*_. On each trial, the drift rate is defined as:

5 $$  v_{t} = v_{\text{mod}} \cdot (Q_{\text{cor},t} - Q_{\text{inc},t}) $$

and within each trial evidence is accumulated as in Eq. 4. Note that, since *v* is defined as the difference of subjective values, the difficulty effect naturally emerges from the model without assuming separate *v* s for easy and difficult choices.

We considered three additional mechanisms and fit different combinations of them, resulting in a total of eight different models. The first variation is similar to one considered for RL models and includes two separate *η*s for positive and negative prediction errors (as in Pedersen et al.,). The second variation is similar to one considered in the DDM to account for the magnitude effect. However, because subjective values are learned in time, instead of fitting separate *a* s for different pairs of options (as we do in the DDM), we propose a trial-by-trial modulating mechanism:

6 $$  a = exp(a_{\text{fix}} + a_{\text{mod}} \cdot \overline{Q}_{\text{pres}}) $$

where *a*_fix_ is the fixed threshold, *a*_mod_ is the threshold modulation parameter, and $\overline {Q}_{\text {pres}}$ is the average subjective value of the presented options. When *a*_mod_ = 0, this model reduces to the simplest model. The third variation is to make the mapping between subjective values and choices in the RLDDM more similar to the mapping in the soft-max choice rule. In Eq. 5, *v* is linearly related to the difference in values. Since different pairs of options can have very similar or very different values (e.g., in Fig. 1, pairs AB and AC), participants might differ in how sensitive they are to these differences. In RL models, this is regulated by the sensitivity parameter *𝜃*. We therefore propose a very similar, nonlinear transformation of the value differences in the definition of *v*:

7 $$  v_{t} = S\left( v_{\text{mod}} \cdot (Q_{\text{cor},t} - Q_{\text{inc},t})\right), $$

with

8 $$  S(z) = \frac{2 \cdot v_{\max}}{1 + e^{-z}} - v_{\max} $$

where *S*(*z*) is an S-shaped function centered at 0, and *v*_max_ is the maximum absolute value that *S*(*z*) can take on: $\lim _{z\to \pm \infty } S(z) = \pm v_{\max }$. While *v*_max_ only affects the maximum and minimum values that the drift rate can take, *v*_mod_ affects the curvature of the function. Smaller values of *v*_mod_ lead to more linear mapping between the value difference and the drift rate, and therefore less sensitivity to value differences. Note that this model only resembles the previous models in the limit (i.e., when *v*_max_ has higher values).

### Analysis of the behavioral effects

To assess the difficulty and the magnitude effects, we fit two separate Bayesian hierarchical models: a logistic regression on accuracy and a linear regression on log-transformed RTs. Accuracy was coded as 0 if the option with the lower mean reward was chosen (e.g., A is chosen over B), and as 1 if the option with higher mean reward was chosen (e.g., B is chosen over A). For both models, we included magnitude and difficulty as predictors and tested main effects and the interaction. Magnitude was defined as the true mean reward in each pair of options, and was standardized before fitting. Easy trials were coded as 1 and difficult trials as -1. For simplicity, and because we were interested in cross-trial effects, even though we were dealing with time-series data, no information about trial number was included in the models.

All models were fit using PyStan 2.18, a Python interface to Stan (Carpenter et al., 2017). We ran four parallel chains for 8000 iterations each. The first halves of each chain were warm-up samples and were discarded. To assess convergence, we computed the Gelman-Rubin convergence diagnostic $\hat {R}$ (Gelman & Rubin, 1992). As an $\hat {R}$ close to 1 indicates convergence, we considered a model successfully converged when $\hat {R}\leq 1.01$. Weakly informative priors were chosen for both models. For a graphical representation of the Bayesian hierarchical models and for the exact prior distributions, see Appendix A.

To assess whether difficulty and magnitude had an effect on the behavioral data, we calculated the 95% Bayesian credible interval (BCI) on the posterior mean group distribution of the regression coefficients. If the BCI included 0, we concluded that there was no effect of a manipulation on either RT or choices. Finally, to assess model fit, we computed posterior predictive checks (Gelman, Meng, & Stern, 1996) for mean accuracy and mean RT for each pair of options and looked whether the 95% BCIs of the posterior predictive distributions included the observed mean accuracies and RTs for AB, AC, BD, and CD. Posterior predictive distributions are useful to assess the quality of the models in their ability to predict patterns observed in the data. To approximate the posterior predictive distributions, we drew 500 samples from the posterior distribution, generated 500 independent datasets, and then computed the mean accuracy and mean RTs in each dataset, separately for choice pairs.

### Model fitting and model comparison

For all classes of cognitive models, parameters were estimated using a Bayesian hierarchical modeling approach. Again, all models were fit using PyStan. Since the models vary in their complexity, the sampler was run for a different number of iterations. We first started with few samples (i.e., 1000) and checked for convergence, reflected in $\hat {R}\leq 1.01$. If the model did not converge, more samples were collected. We also checked for saturation of the maximum tree depth (considered satisfactory if less than .1%), energy Bayesian Fraction of Missing Information, and for divergences (considered satisfactory if less than .1%). Four parallel chains were run for all models and only the second half of each chain was kept for later analyses.

To assess the predictive accuracy of the models, we computed the widely applicable information criterion (WAIC; Watanabe, 2013). To compute the WAIC, we used the variance of individual terms in the log predictive density summed over the data points to correct for model complexity, as it approximates best the results of leave-one-out cross-validation (Gelman, Carlin, Stern, & Rubin, 2014). We also computed the standard error of the difference in the predictive accuracy of the best RLDDM, DDM, and among the models of Pedersen et al.,, using the R package loo (Vehtari, Gelman, & Gabry, 2017). This measure provides a better understanding of the uncertainty around the difference in WAIC scores. We then proceeded with the posterior predictive checks: Posterior predictives were calculated for mean accuracy and mean RT across learning by binning the trials within the learning blocks in eight groups of ten trials and across the pairs of options AB, AC, BD, and CD. As for the regression analyses, we sampled 500 parameter sets from the joint posterior distribution and generated 500 independent full datasets using those parameters. We then computed the mean accuracy and RTs in each dataset, separately for choice pairs and trial bins.

For a graphical representation of the Bayesian hierarchical models, and details about the prior distributions, see Appendix A. It has been shown that RL models can suffer from poor identifiability due to low information content in the data (Spektor & Kellen, 2018). To alleviate this concern, we conducted a parameter recovery study whose results can be found in Appendix D.

## Results

Five participants were excluded for not reaching the minimum criterion of accuracy (see Methods section), so that the data of 27 participants were included in the following analyses. The mean accuracy ranged from .43 to .53 (*M* = .49, *SD* = .04) for the excluded participants, and from .62 to .94 (*M* = .81, *SD* = .08) for the remaining ones.

### Behavioral results

**Fig. 3:**
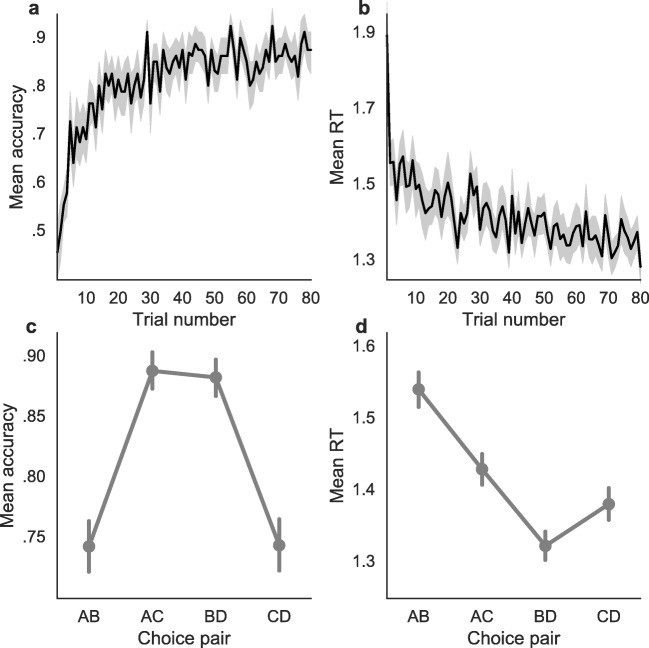
Mean accuracy (**a**) and RT (**b**) across participants as it develops throughout learning. *Solid lines* represent the mean across experimental blocks and participants, while the *shaded areas* represent 95% confidence intervals. Mean accuracy (**c**) and RT (**d**) across participants and for different pairs of options. Choices between options AC and BD were easier than between AB and CD, while the mean reward was highest in pair CD followed by BD, AC, and AB. The *dots* represent the mean across trials, while the *bars* represent 95% confidence intervals. Multilevel bootstrap was performed to account for repeated measures and therefore individual variability

On average, participants showed substantial learning effects (Fig. 3a and b): The higher-valued option was chosen more often throughout the trials (from *M* = .71 in the first 20 trials, to *M* = .86 in the last 20 trials), while at the same time responses became faster (from *M* = 1.51 s in the first 20 trials, to *M* = 1.36 s in the last 20 trials). They also showed difficulty and magnitude effects (Fig. 3c and d): They were more accurate in easier compared to difficult choices (*M* = .89 compared to *M* = .74), while at the same time being faster (*M* = 1.38 s compared to *M* = 1.46 s); they were not more accurate in higher valued choice pairs compared to lower valued ones (*M* = .81 compared to *M* = .81), but they were faster (*M* = 1.35 s compared to *M* = 1.48 s).

**Fig. 4:**
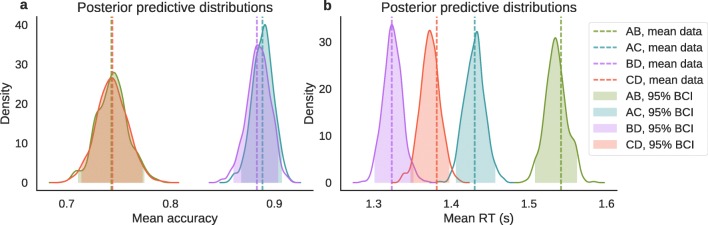
Posterior predictive distributions of mean accuracy (**a**) and mean RT (**b**) for different option pairs according to the logistic and linear regression models. The mean data (*dotted lines*) are compared to the regression model predictions (*solid lines*). The *shaded areas* represent the 95% Bayesian credible interval (BCI) of the posterior predictive distributions. Pairs AB and CD have similar mean values while pairs AC and BD have different mean value. Mean values increase from options AB, to AC, BD and CD

To test difficulty and magnitude effects on accuracy and RTs across trials, we fit two regression models. Results from the logistic regression model on accuracy suggest that only difficulty, but not magnitude, had an effect on accuracy. There was no interaction between difficulty and magnitude on accuracy. In particular, participants were less accurate when choosing between AB and CD compared to AC and BD. The 95% BCI was higher than 0 (0.39 to 0.71, *M* = 0.56) for the mean group difficulty coefficient (meaning that easier decisions were more accurate), but it was around 0 for the magnitude coefficient (−0.27 to 0.16, *M* = − 0.05) and for the interaction coefficient (−0.26 to 0.15, *M* = − 0.05). To check whether the regression model predictions fit the data well, we used posterior predictive checks. In particular, we checked whether the regression model correctly predicts the mean accuracy across different pairs of options. As can be seen in Fig. 4a, the regression model correctly predicts the observed pattern.

Results from the linear regression model on RTs suggest that both magnitude and difficulty as well as their interaction had an effect on RTs. In particular, participants responded faster when BD and CD were presented, compared to AB and AC. They were also faster in easy trials (pairs AC and BD) compared to difficult trials (pairs AB and CD) and this effect was stronger for less attractive options. The 95% BCI was lower than 0 for the group-level magnitude coefficient (−0.12 to −0.07, *M* = − 0.10), for the difficulty coefficient (−0.04 to −0.02, *M* = − 0.03) and for the interaction coefficient (−0.06 to −0.02, *M* = − 0.04). Similarly to the previous regression model, we also checked whether the regression model correctly predicts the mean RTs across the different pairs of options. As can be seen in Fig. 4b, the regression model correctly predicts the observed pattern.

**Table 1:** Widely applicable information criteria of the reinforcement learning models

Model	*η*	*𝜃*	*p*_WAIC_	−lppd	WAIC
RL 1	One	Fixed	48	2636	5368
RL 2	One	Power	45	2645	5381
RL 3	Two	Fixed	63	2569	5265
RL4	Two	Power	72	2573	5291

*Note*. Models 1 to 4 are reinforcement learning (RL) models with learning rate *η* and sensitivity *𝜃*. The models could have a single or separate *η* (for positive and negative prediction errors). *𝜃* could be fixed in time or increase as a power function of the number of times an option was seen. *p*_WAIC_ is the effective number of parameters, lppd is the log predictive accuracy of the fitted model to data, and WAIC is the information criterion. Lower WAICs indicate better fits to data after accounting for model complexity.

### Cognitive modeling

To better understand the learning and decision processes underlying value-based decisions, we fit and compared three different classes of models: RL models, the DDM, and RLDDMs, as well as previous attempts of combining RL and the DDM (Pedersen et al., 2017). While RL models can be only fit to choices, the DDM and RLDDM can be simultaneously fit to choices and RTs. However, the DDM does not take trial-by-trial information, such as the reward feedback, into account. In the following section, we report results from the model fitting and model comparison of these models.

**Fig. 5:**
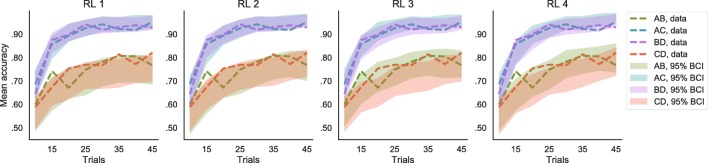
Posterior predictive distributions of mean accuracy according to the reinforcement learning (RL) models. The data (*dotted lines*) are compared to the 95% Bayesian credible interval (BCI) of the posterior predictive distribution (*shaded areas*), separately for the different options pairs and for eight bins of trials within the learning blocks. Model 1 is the simplest RL model with one learning rate *η* and one sensitivity parameter *𝜃*. Models 3 and 4 have separate *η* for positive and negative prediction errors. In models 2 and 4, *𝜃* increases as a power function of the number of times an option is seen. According to the WAIC the best model is model 3

Among the RL models, model 3 provided the most parsimonious account of the data. This model assumes separate learning rate parameters for positive and negative prediction errors and has a fixed sensitivity parameter throughout learning. Compared to the other models (Table 1), this model had the best predictive accuracy, as indicated by a higher log pointwise predictive density (lppd), and had lower complexity compared to the full model (i.e., model 4), as indicated by the *p*_WAIC_. Judging by the WAIC, models 2 and 4, having an increasing sensitivity in time, did not outperform models 1 and 3, while the separate learning rates increased fitness of the models. This can be further assessed by looking at the 95% BCI of the posterior predictive distribution of mean accuracy across learning and pairs of options (Fig. 5). All models predicted a nonlinear increase in performance throughout the trials, and a difference between easy (i.e., AC and BD) and difficult (i.e., AB and CD) choices.

**Fig. 6:**
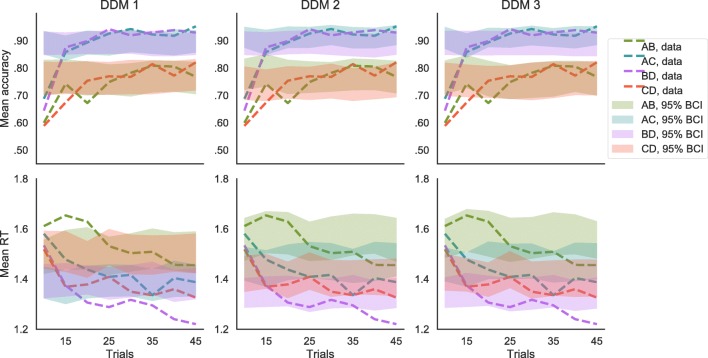
Posterior predictive distributions of mean accuracy and response time (RT) according to the diffusion decision model (DDM). The data (*dotted lines*) are compared to the 95% Bayesian credible interval (BCI) of the posterior predictive distribution (*shaded areas*), separately for the different options pairs and for eight bins of trials within the learning blocks. Models 1 and 2 have separate drift rates *v* for easy and difficult decisions, while model 3 has separate *v* for each option pair. Model 1 has a fixed threshold *a*, while models 2 and 3 have separate *a* for each option pair. According to the WAIC the best model among DDM is model 2

The most parsimonious DDM was model 2, with separate drift rates for easy (i.e., AC and BD) and difficult (i.e., AB and CD) trials and separate response thresholds for pairs of options with different mean reward distributions (i.e., one for each pair: AB, AC, BD, and CD). As shown in Table 2, this model had lower predictive accuracy than model 3, as indicated by the lppd, but had also lower complexity than model 3, as indicated by the *p*_WAIC_. The WAIC was lower for model 2 than for model 3, indicating that model 3 could not compensate its higher complexity with a better fit. Checking the posterior predictives in Fig. 6, we can see that: (a) all three versions of the DDM did not predict any learning effect (i.e., both accuracy and RT are stable across trials); (b) only the versions of the DDM with separate thresholds for different option pairs could predict the magnitude effect on RTs, without changing accuracy predictions (i.e., having lower thresholds, responses in higher-valued pairs are not less accurate but only faster); (c) all models could predict difficulty effects on accuracy and RTs; (d) predictions from model 3 were not qualitatively better than predictions from model 2.

**Table 2:** Widely applicable information criteria of the diffusion models

Model	*v*	*a*	*p*_WAIC_	−lppd	WAIC
DDM 1	Difficulty	One	125	5194	10639
DDM 2	Difficulty	All choice pairs	183	5034	10433
DDM 3	All choice pairs	All choice pairs	232	4986	10435

*Note*. Models 1 to 3 are diffusion decision models (DDMs) with drift rate *v*, decision-threshold *a*, and non-decision time *T*_*er*_. *v* could depend either on choice difficulty only or different *v* could be fitted for each choice pair. *a* could be either fixed across conditions, or separate *a* could be fit for separate pairs of options

**Fig. 7:**
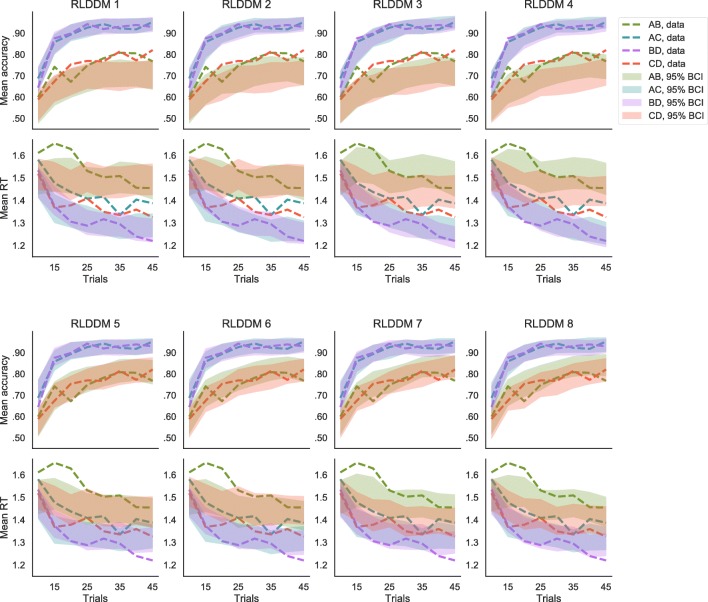
Posterior predictive distributions of mean accuracy and response time (RT) according to the reinforcement learning diffusion decision models (RLDDM). The data (*dotted lines*) are compared to the 95% Bayesian credible interval (BCI) of the posterior predictive distribution (*shaded areas*), separately for the different options pairs and for eight bins of trials within the learning blocks. Models 1 to 4 have a linear mapping between differences in values and the drift rate *v*, and models 5 to 8 have a non-linear mapping. All models with even number have separate learning rate *η* for positive and negative prediction errors. Models 1, 2, 5, and 6 have a fixed threshold *a*, while, in models 3, 4, 7, and 8, *a* is modulated by the average value of the options. According to the WAIC the best model among DDM, RLDDM and the models of Pedersen et al., (2017), is model 8

Among our proposed RLDDMs, the most parsimonious model was the last, full model. In this model, separate learning rates were fit for positive and negative prediction errors, the drift rate was an S-shaped function of the difference in subjective values, and the threshold was modulated by the learned average subjective value of the presented options, so that the threshold was lower when the expected reward was higher. As shown in Table 3, this model had highest predictive accuracy, as indicated by the lppd, and highest complexity, as indicated by the *p*_WAIC_. Having the lowest WAIC suggests that the model’s complexity is compensated by its superior fit to the data. In Fig. 7, posterior predictives reveal how the different models were able to capture the observed patterns in accuracy and RTs. In particular: (a) all models were able to capture learning effects as a decrease in RTs and increase in accuracy over time; (b) all models captured difficulty effects, but only models 5 to 8, by including a non-linear mapping between value differences and drift rate, did not underestimate accuracy for difficult (i.e., AB and CD) decisions; (c) only the models that included a modulating effect of values on the decision threshold could capture the magnitude effect on RTs. While no significant qualitative pattern could be observed for two compared to one learning-rate models, all models with two learning rates had slightly lower WAICs compared to their analogues with only one learning rate. The best RLDDM also outperformed the best DDM, both in terms of WAIC and in terms of posterior predictive checks.

**Table 3:** Widely applicable information criteria of the reinforcement learning diffusion decision models

Model	*η*	*v*	*a*	*p*_WAIC_	−lppd	WAIC
RLDDM 1	One	Linear	Fixed	111	5129	10481
RLDDM 2	Two	Linear	Fixed	134	5051	10369
RLDDM 3	One	Linear	Modulated	145	4942	10174
RLDDM 4	Two	Linear	Modulated	159	4866	10048
RLDDM 5	One	Sigmoid	Fixed	137	4930	10135
RLDDM 6	Two	Sigmoid	Fixed	159	4861	10039
RLDDM 7	One	Sigmoid	Modulated	164	4672	9672
RLDDM 8	Two	Sigmoid	Modulated	190	4613	9607

*Note*. Models 1 to 8 are reinforcement learning diffusion decision models (RLDDMs) with learning rate *η*, decision threshold *a*, and non-decision time *T*_*er*_. The models could have a single or separate *η* (for positive and negative prediction errors), linear or non-linear mapping of value differences to *v*, and fixed or value-modulated *a*

**Table 4:** Widely applicable information criteria of Pedersen et al., (2017)’s models

Model	*η*	*v*	*a*	*p*_WAIC_	−lppd	WAIC
Pedersen RLDDM 1	One	Fixed	Power	118	5100	10436
Pedersen RLDDM 2	One	Power	Power	112	5326	10875
Pedersen RLDDM 3	Two	Fixed	Power	141	5020	10322
Pedersen RLDDM 4	Two	Power	Power	126	5240	10732

*Note*. Models 1 to 4 are Pedersen et al., (2017) best fitting reinforcement learning diffusion decision models (RLDDMs) with learning rate *η*, decision threshold *a*, and non-decision time *T*_*er*_. The models could have a single or separate *η* (for positive and negative prediction errors), fixed or increasing *v*, and fixed or decreasing *a*

**Fig. 8:**
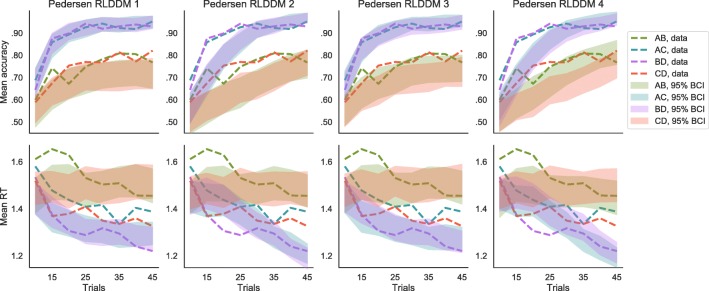
Posterior predictive distributions of mean accuracy and response time (RT) according to Pedersen et al., (2017) best-fitting models. The data (*dotted lines*) are compared to the 95% Bayesian credible interval (BCI) of the posterior predictive distribution (*shaded areas*), separately for the different options pairs and for eight bins of trials within the learning blocks. Models 1 and 3 have a linear scaling parameter for the drift rate *v*, while in models 2 and 4 this parameter increases as a power function of the trial number. Models 3 and 4 have separate learning rate *η* for positive and negative prediction errors. In all models, the threshold *a* decreases as a power function of the number of trials. According to the WAIC, the best model among the models of Pedersen et al., (2017) is model 3

Among Pedersen et al.,’s (2017) models, the most parsimonious one was a model with separate learning rates for positive and negative reward prediction errors, a drift rate that is linearly proportional to the difference in values of the correct and incorrect options, and a threshold that decreases as a power function of time within a block. Note that this was also the most parsimonious model in their task. A quantitative comparison between the different combinations of models can be found in Table 4, while posterior predictives can be seen in Fig. 8. The best of these models neither outperformed any of those RLDDMs that included the S-shaped mapping function in the drift rate, nor the ones having a modulating mechanism of value for the threshold.

Lastly, to have a measure of uncertainty of the difference in WAIC scores, we calculated the standard error of the difference in predictive accuracy of the best fitting RLDDM with the best fitting DDM, finding a substantial difference between the scores (*elpd*_diff_ = − 415.4, *SE* = 38.3), and the best fitting model of Pedersen et al.,, finding a substantial difference between the scores (*elpd*_diff_ = − 255.1, *SE* = 33.1).

## Discussion

In the present work, we proposed a new process model for value-based decision-making during learning. To test this model, we collected data from participants performing a multi-armed bandit task, in which both the value difference between options as well as the mean reward of different pairs of options were manipulated. This was done to elicit two value-based behavioral effects known in the literature: the difficulty (e.g., Dutilh and Rieskamp, 2016; Oud et al., 2016; Polania et al., 2014) and the magnitude (e.g., Palminteri et al., 2015; Pirrone et al., 2017; Polania et al., 2014) effects. We first assessed value effects across all trials by fitting regression models on accuracy and RTs. We observed a magnitude effect on RTs only and a difficulty effect on both RTs and choices. To gain insights into the separate learning and value mechanisms, we tested our model against RL models (from the value-based decision-making tradition) and standard DDMs (from the perceptual decision-making tradition). We also compared our model to a previously proposed class of combined RLDDM (Pedersen et al., 2017). Different classes of models were tested, when possible, quantitatively (i.e., whether our model provided a better account of the data using a relative measure) and qualitatively (i.e., whether our model captured the observed patterns that were related to the experimental manipulations).

**Fig. 9:**
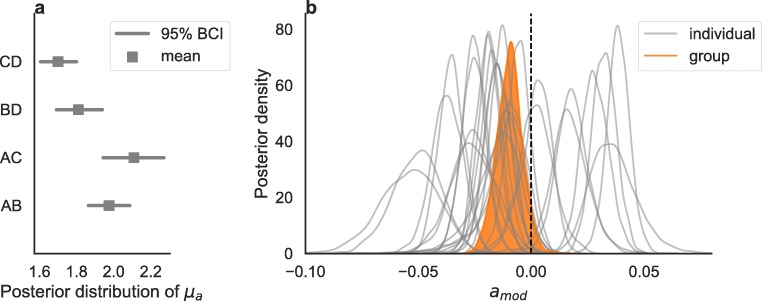
**a** Posterior distributions of the mean group threshold parameters *μ*_*a*_ of the diffusion decision model in which separate drift rates were fit for easy and difficult decisions, and separate thresholds were fit for all different pairs of choices. *Solid lines* represent the 95% Bayesian credible interval, and squares represent the mean of the posterior distribution. **b** Posterior distribution of the mean group (*in orange*) and of the individual (*in grey*) threshold modulator parameters *a*_mod_ of the full reinforcement learning diffusion decision model

Our analyses suggest that, while difficulty has an effect on both accuracy and RTs, magnitude only affects RTs: Difficult decisions tend to be slower and less accurate, while decisions among higher-valued options tend to be faster, but not less accurate. These results confirm previous studies that investigated value-based decisions after preferences have been formed (e.g., Pirrone et al., 2017; Polania et al., 2014) as well as studies that compared approach and avoidance behavior (e.g., Cavanagh, Wiecki, Kochar, & Frank, 2014; Palminteri et al., 2015). In line with previous studies, we also found that participants tended to become faster and more accurate during learning, and more so for easy compared to difficult trials. These behavioral patterns (a) can only partially be predicted by RL models, as they do not predict RTs, (b) are not predicted by the DDM, as it does not take trial-by-trial feedback into account, and (c) are fully predicted by RLDDM. By presenting easy and difficult pairs of options, we also showed that a nonlinear mapping between the difference in subjective values (learned via RL) and the DDM drift rate improved the model fit substantially. In other words, the drift rate may not double for option pairs whose difference of means is twice as large (see Teodorescu, Moran, & Usher, 2015 for a similar finding in perceptual decisions). As a consequence, models that do not assume a nonlinear mapping tend to underestimate the accuracy in the difficult trials. Finally, to give an account of the magnitude effect during learning, we proposed a mechanism in which the threshold is modulated by the mean subjective values of the presented options. By having a lower decision threshold, decisions between higher-valued pairs of options become faster, while accuracy only decreases to a minor extent. This mechanism is also suggested by the estimated thresholds in the DDM, separately fit for each pair of options: Higher-valued pairs have a lower threshold (see Fig. 9a). In the RLDDM, this is obtained by a negative threshold modulation parameter: Negative values imply a lower threshold for higher-valued options (see Fig. 9b). Cavanagh, Wiecki, Kochar, and Frank (2014) also reported a reduction of the threshold when comparing approach to avoidance behavior, and interpreted this finding as a facilitation effect on the cortico-striatal indirect pathway due to increased dopamine levels, based on previous work (Wiecki & Frank, 2013). In both RL and RLDDM models, separating the learning rate for positive and negative prediction errors increased the predictive accuracy of the models, as indicated by a lower WAIC. Although a qualitative difference in fit cannot be visualized in the posterior predictive checks we calculated, this result is in line with previous research (Gershman, 2015; Lefebvre et al., 2017).

Notably, our proposed model has stricter constraints than the DDM: When fitting the DDM to behavioral data, all trials are collapsed into two RT distributions for choosing high- and low-value options, meaning that slower decisions will be in the right tail of the distribution, independently of their occurrence at the beginning or at the end of a learning block. In the RLDDM, the trial order is taken into account, as the drift rate and the threshold depend on the learned values in each trial. When fitting the DDM using different parameters per condition, we also have less constraints. By explicitly relating the difference in values to the drift rate, and the mean learned values to the threshold, we propose mapping functions that can accommodate the observed results, providing more mechanistic explanations.

We also compared our best RLDDM to previously proposed RLDDMs. We fit the four best models proposed by Pedersen et al., (2017), and compared them quantitatively (see Table 4) and qualitatively (see Fig. 8) to our models. The quantitative comparison confirmed that our best model had better predictive accuracy than those previous models. The qualitative comparison shows that the models proposed by Pedersen et al., (2017), which assume a linear mapping between value differences and drift rate, largely overestimate the difference in performance between easy and difficult choices. Models that assumed an increasing scaling parameter for the drift rate (i.e., models 2 and 4 in Fig. 8) predicted an almost linear increase in accuracy throughout time, while the data suggest a more asymptotic learning curve. Moreover, all models proposed by Pedersen et al., (2017) predicted that the RTs for difficult (i.e., AB and CD) decisions do not decrease in time as much as it was observed in the data. Because Pedersen et al., (2017) did not show the development of mean RT throughout learning, we cannot assess whether this discrepancy was also present in their data. Finally, since Pedersen et al., (2017) did not manipulate mean reward of pairs of options and did not include a mechanism to account for the magnitude effect, their model is unable to explain this effect.

In this study, by combining DDM and RL models, we aimed at providing a psychological account of the processes underlying behavior in a RL paradigm, where the expected reward and the difficulty varies across trials. As behavioral effects in this task are not necessarily evident in all behavioral measures (in particular, the magnitude effect is only present in RTs), we showed how, by simultaneously fitting choices and RTs and constraining them by feedback data, we can provide a more complete account of the cognitive processes in this task and identify mechanisms that would remain undetected if only choices but not RTs were taken into account.

Future work should test whether the best RLDDM is also successful in describing behavior in different learning paradigms by, for instance, investigating the magnitude effect in the presence of gains and losses or by manipulating the dispersion of the reward distributions. Moreover, RLDDMs could be validated by linking model parameters to neural measures. Trial-by-trial variables such as prediction errors, reward expectation signals, trial-by-trial threshold modulations, and individual parameters such as learning rates for negative and positive prediction errors can be easily estimated using this model. It would be interesting to see whether the model predictions about prediction errors are in line with previous work in RL neuroscience (O’Doherty, Hampton, & Kim, 2007), and whether the trial-by-trial adjustments of decision thresholds are mapped onto the same brain circuitry which has been reported for decision-making paradigms without learning (Gluth & Rieskamp, 2017; Gluth et al., 2012; van Maanen et al., 2011). An obvious limitation of any RLDDM is that it can only be fit to two-alternative forced choice tasks. This is problematic for paradigms such as the Iowa gambling task (Bechara et al., 1994), for example, in which participants choose between four decks in each trial, or for studying context effects in experience-based decisions with more than two options (Spektor, Gluth, Fontanesi, & Rieskamp, in press). A different, multi-alternative SSM that is, for instance, based on the linear ballistic accumulator model (Brown & Heathcote, 2008) could offer an alternative.

In conclusion, by integrating knowledge from two separate research traditions, we showed how an extended computational model can promote our understanding of the cognitive processes underlying value-based decisions during learning.

## Figures and Tables

**Fig. 10:**
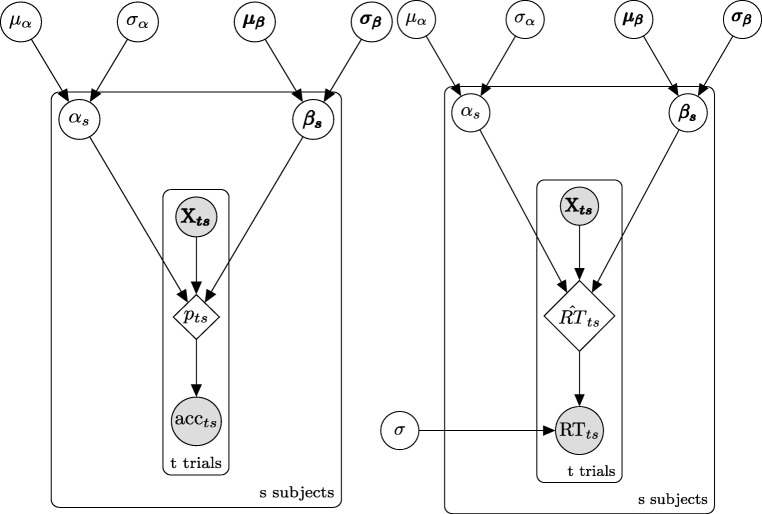
Graphical representation of the Bayesian hierarchical linear regression (on the *left*) and logistic regression (on the *right*) models. *Shaded nodes* represent observed variables, while *non-shaded nodes* represent latent variables. *Squared nodes* represent deterministic variables

**Fig. 11:**
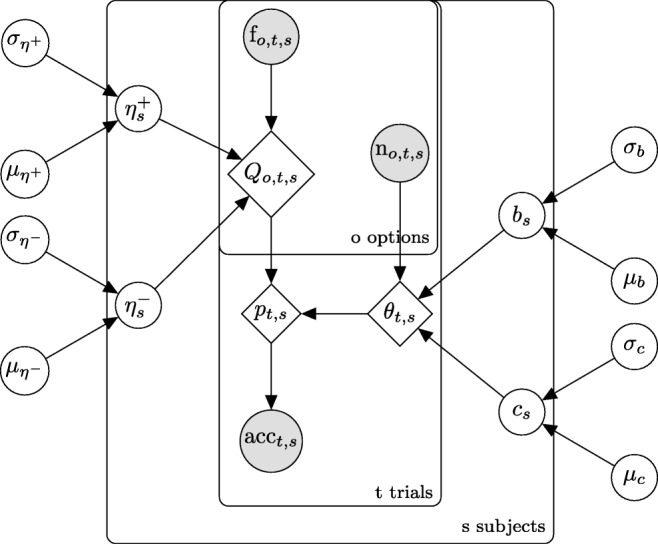
Graphical representation of the Bayesian hierarchical full reinforcement learning (RL) model. *Shaded nodes* represent observed variables, while *non-shaded nodes* represent latent variables. *Squared nodes* represent deterministic variables

**Fig. 12:**
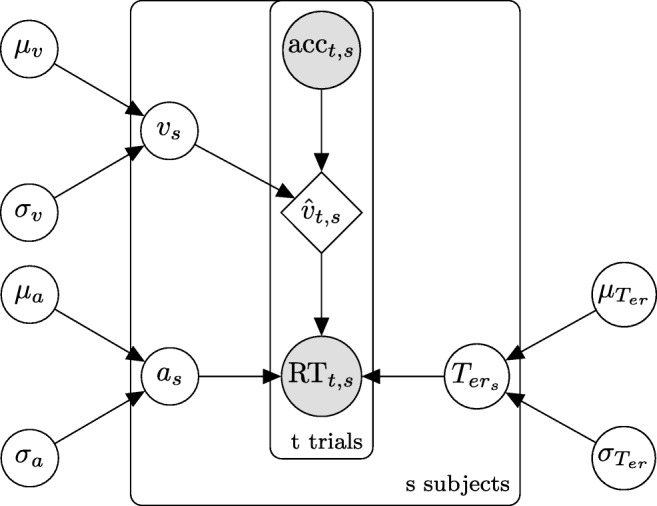
Graphical representation of the Bayesian hierarchical diffusion decision model (DDM). *Shaded nodes* represent observed variables, while *non-shaded nodes* represent latent variables. *Squared nodes* represent deterministic variables

**Fig. 13:**
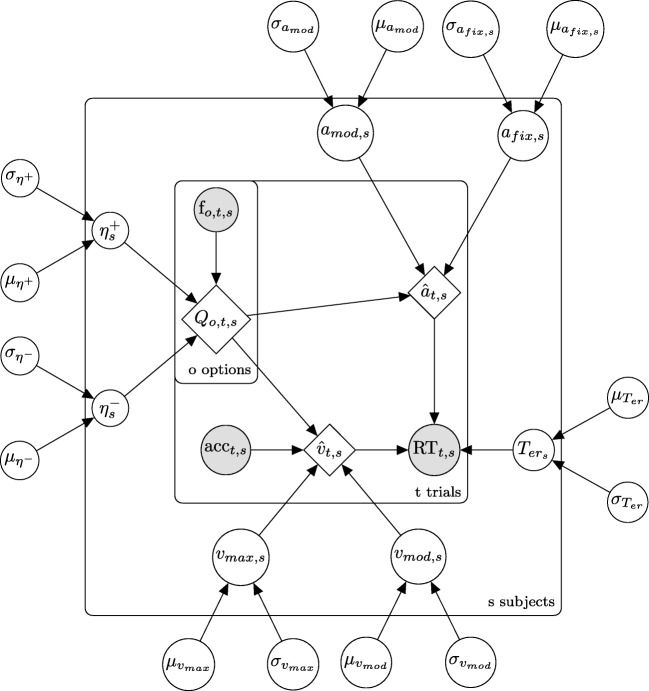
Graphical representation of the Bayesian hierarchical full reinforcement learning diffusion decision model (RLDDM). *Shaded nodes* represent observed variables, while *non-shaded nodes* represent latent variables. *Squared nodes* represent deterministic variables. Vectors are represented in *bold font*

**Fig. 14:**
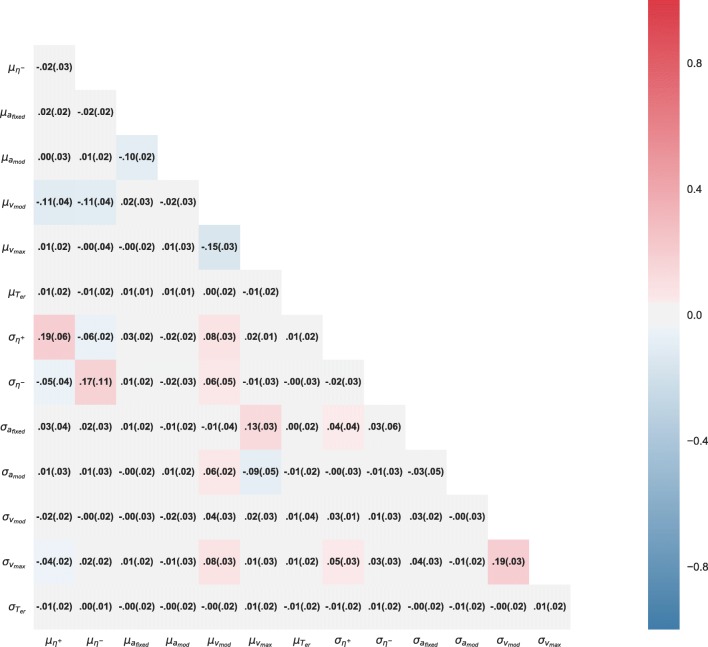
Mean (and, between brackets, SD) of the correlation between the samples of the group-level parameters, across simulations

**Fig. 15:**
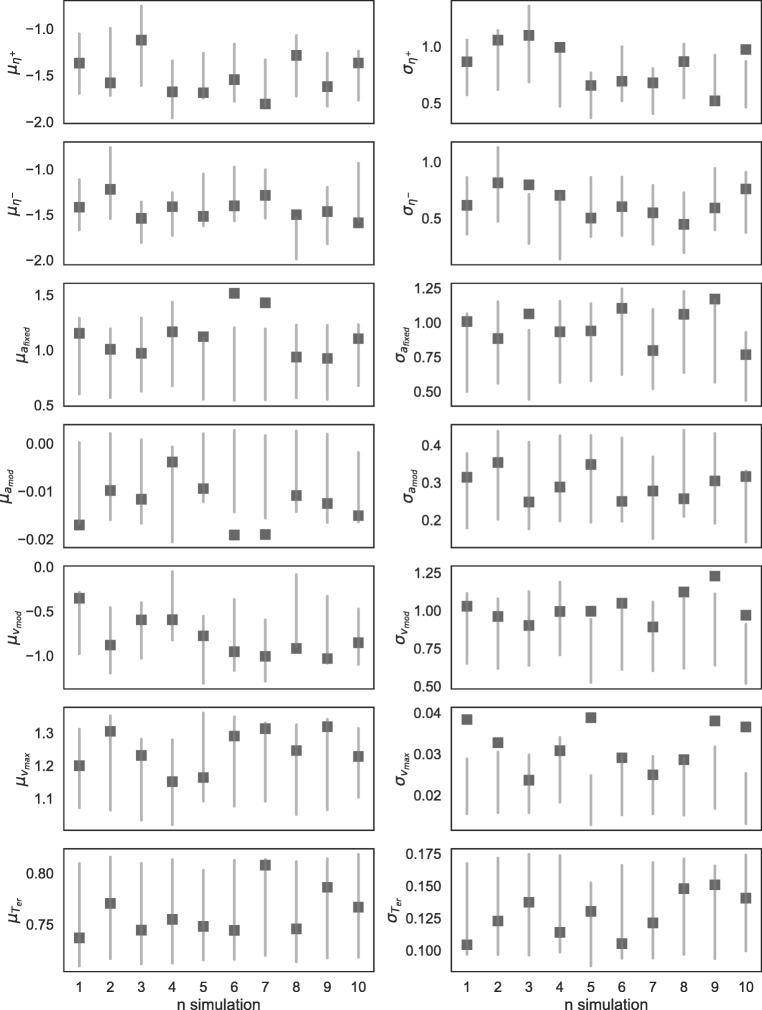
95% BCI of the estimated mean and standard deviation of the group parameter distributions (*grey lines*) and true generating group parameters (*grey squared dots*)

**Fig. 16:**
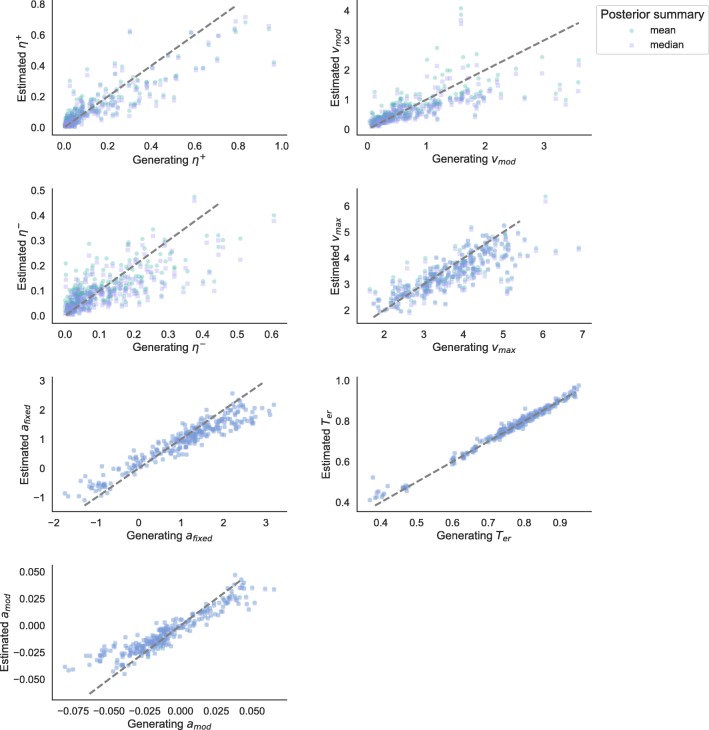
Scatter plot of the estimated individual parameters, summarized as mean and median of the posterior distribution, against the true generating parameters. All ten simulations are plotted here together

## Appendix: Appendix A: Bayesian hierarchical regression models

A graphical representation of the linear regression model of accuracy can be seen in Fig. 10 on the right. The model, model priors, and hyper-priors were specified as:

$$\mu_{\alpha} \sim \mathcal{N}(0, 5); \sigma_{\alpha} \sim \mathcal{HN}(0, 5)$$

$$\pmb{\mu_{\beta}} \sim \mathcal{N}(0, 5); \pmb{\sigma_{\beta}} \sim \mathcal{HN}(0, 5)$$

$$\alpha \sim \mathcal{N}(\mu_{\alpha}, \sigma_{\alpha}); \beta \sim \mathcal{N}(\mu_{\beta}, \sigma_{\beta})$$

$$p_{t,s} = logit({\text{X}_{t,s} \beta_{s}} + \alpha_{s}); \text{acc}_{t,s} \sim \operatorname{Bern}(p_{t,s})$$

where *α* and *β* are, respectively, the intercept and the vector of coefficients, and **X** is the predictors matrix. $\mathcal {N}$ is a normal distribution with parameters mean and standard deviation, $\mathcal {HN}$ is a half-normal distribution with parameters mean and standard deviation, and Bern is a Bernoulli distribution with a success probability parameter.

A graphical representation of the linear regression model of RT can be seen in Fig. 10 on the left. The model, model priors, and hyper-priors were specified as:

$$\mu_{\alpha} \sim \mathcal{N}(0, 5); \sigma_{\alpha} \sim \mathcal{HN}(0, 5)$$

$$\pmb{\mu_{\beta}} \sim \mathcal{N}(0, 5); \pmb{\sigma_{\beta}} \sim \mathcal{HN}(0, 5)$$

$$\sigma \sim \mathcal{HN}(0, 5)$$

$$\alpha \sim \mathcal{N}(\mu_{\alpha}, \sigma_{\alpha}); \beta \sim \mathcal{N}(\mu_{\beta}, \sigma_{\beta})$$

$$ \hat{\text{RT}}_{t,s} = {\text{X}_{t,s}\beta_{s}} + \alpha_{s}; \text{RT}_{t,s} \sim \mathcal{N}(\hat{\text{RT}}_{t,s}, \sigma) $$

where *α* and *β* are, respectively, the intercept and the vector of coefficients, *σ* is the noise term, and **X** is the predictors matrix. $\mathcal {N}$ is a normal distribution with parameters mean and standard deviation, and $\mathcal {HN}$ is a half-normal distribution with parameters mean and standard deviation.

Models specification followed the approach of Gelman et al., (2014), while priors followed the prior choice recommendations in the Stan manual.

## Appendix B: Bayesian hierarchical cognitive models

A graphical representation of the full reinforcement learning model (i.e., with separate learning rates *η* for positive and negative prediction error and increasing sensitivity *𝜃*) can be seen in Fig. 11. The model, model priors, and hyper-priors were specified as:

$$\mu_{\eta^{\pm}} \sim \mathcal{N}(0, .8); \sigma_{\eta^{\pm}} \sim \mathcal{HN}(0, .5)$$

$$\eta^{\pm} \sim \phi(\mathcal{N}(\mu_{\eta^{\pm}}, \sigma_{\eta^{\pm}}))$$

$$\mu_{b} \sim \mathcal{N}(5, .5); \sigma_{b} \sim \mathcal{HN}(0, .5)$$

$$ b \sim exp(\mathcal{N}(\mu_{b}, \sigma_{b})) $$

$$\mu_{c} \sim \mathcal{N}(-.5, .5); \sigma_{c} \sim \mathcal{HN}(0, .5)$$

$$c \sim exp(\mathcal{N}(\mu_{c}, \sigma_{c}))$$

$$\text{acc}_{t,s} \sim \operatorname{Bern}(logit(p_{t,s}))$$

where *η*^±^ are the learning rates for positive and negative prediction errors, *b* and *c* are the parameters that define the increase of *𝜃* in time (see Eq. 3). $\mathcal {N}$ is a normal distribution with parameters mean and standard deviation, $\mathcal {HN}$ is a half-normal distribution with parameters mean and standard deviation, and Bern is a Bernoulli distribution with a success probability parameter.

**Table 5:** Group parameter estimates of the full reinforcement learning model

Parameter	*M*	*SD*	2.5% percentile	97.5% percentile
$\phi (\mu _{\eta ^{+}})$	0.07	0.02	0.04	0.10
$\sigma _{\eta ^{+}}$	0.57	0.11	0.38	0.82
$\phi (\mu _{\eta ^{-}})$	0.24	0.04	0.17	0.34
$\sigma _{\eta ^{-}}$	0.32	0.21	0.02	0.79
$exp(\mu _{\theta })$	0.35	0.05	0.26	0.48
$\sigma _{\theta }$	0.63	0.12	0.44	0.88

*Note*. The best-fitting reinforcement model had separate learning rates *η*^+^ and *η*^−^ for positive and negative prediction errors, and fixed sensitivity *𝜃* throughout learning. Note that $\mu _{\eta ^{+}}$, $\mu _{\eta ^{-}}$, and *μ*_*𝜃*_ were transformed for interpretability

**Table 6:** Group parameter estimates of the diffusion decision model

Parameter	*M*	*SD*	2.5% percentile	97.5% percentile
$\mu _{v, \text {diff}}$	0.67	0.07	0.54	0.81
$\sigma _{v, \text {diff}}$	0.33	0.05	0.24	0.45
$\mu _{v, \text {easy}}$	1.28	0.08	1.11	1.44
$\sigma _{v, \text {easy}}$	0.42	0.07	0.31	0.58
$\mu _{a,\text {AB}}$	1.97	0.06	1.86	2.09
$\sigma _{a,\text {AB}}$	0.27	0.05	0.19	0.37
$\mu _{a,\text {AC}}$	2.11	0.08	1.94	2.27
$\sigma _{a,\text {AC}}$	0.39	0.07	0.28	0.54
$\mu _{a,\text {BD}}$	1.81	0.06	1.69	1.94
$\sigma _{a,\text {BD}}$	0.31	0.05	0.22	0.42
$\mu _{a,\text {CD}}$	1.70	0.05	1.61	1.80
$\sigma _{a,\text {CD}}$	0.23	0.04	0.17	0.32
$\mu _{T_{er}}$	0.75	0.02	0.71	0.80
$\sigma _{T_{er}}$	0.13	0.02	0.10	0.17

*Note*. The diffusion decision model had separate drift rate *v* for easy (between AC and BD) and difficult (between AB and CD) choices, a different threshold *a* for each pair of options, and one non-decision time *T*_*e**r*_

A graphical representation of the Bayesian hierarchical diffusion decision model (DDM) can be seen in Fig. 12. The model, model priors, and hyper-priors were specified as:

$$\mu_{v} \sim \mathcal{N}(1, 2); \sigma_{v} \sim \mathcal{HN}(0, 2)$$

$$v \sim \mathcal{N}(\mu_{v}, \sigma_{v})$$

$$\mu_{a} \sim {\Gamma}(1, 2); \sigma_{a} \sim \mathcal{HN}(0, .5)$$

$$ a \sim \mathcal{N}(\mu_{a}, \sigma_{a}) $$

$$\mu_{T_{er}} \sim \mathcal{U}(0, 1); \sigma_{T_{er}} \sim \mathcal{HN}(0, .5)$$

$$T_{er} \sim \mathcal{N}(\mu_{T_{er}}, \sigma_{T_{er}})$$

$$\hat{v}_{t,s}= \left\{\begin{array}{ll} v_{s}, & \text{if } \text{acc}_{t,s}= 1\\ -v_{s}, & \text{if } \text{acc}_{t,s}=-1 \end{array}\right.$$

$$\text{RT}_{t,s} \sim Wiener(\hat{v}_{t,s}, a_{s}, T_{er_{s}}, .5)$$

where *W**i**e**n**e**r* is the Wiener distribution (Navarro and Fuss, 2009) with parameters drift rate, threshold, non-decision time, and relative starting-point, Γ is a gamma distribution with parameters shape and scale, and $\mathcal {U}$ is a uniform distribution with parameters lower and upper bounds. In our case, the starting-point is fixed at .5, since the options were randomly positioned on the right or left of a fixation cross across the trials and we therefore assumed that participants were not biased towards a particular position.

**Table 7:** Group parameter estimates of the full reinforcement learning diffusion decision model

Parameter	*M*	*SD*	2.5% percentile	97.5% percentile
$\phi (\mu _{\eta ^{+}})$	0.07	0.02	0.03	0.12
$\sigma _{\eta ^{+}}$	0.75	0.15	0.50	1.09
$\phi (\mu _{\eta ^{-}})$	0.08	0.02	0.05	0.14
$\sigma _{\eta ^{-}}$	0.58	0.13	0.37	0.87
$exp(\mu _{v_{\text {mod}}})$	0.48	0.10	0.32	0.70
$\sigma _{v_{\text {mod}}}$	0.85	0.14	0.59	1.14
$exp(\mu _{v_{\max }})$	3.47	0.25	2.98	3.98
$\sigma _{v_{\max }}$	0.31	0.07	0.20	0.47
$\mu _{a_{\text {fixed}}}$	1.00	0.20	0.62	1.39
$\sigma _{a_{\text {fixed}}}$	0.97	0.14	0.73	1.26
$\mu _{a_{\text {mod}}}$	−0.010	0.006	−0.021	0.001
$\sigma _{a_{\text {mod}}}$	0.027	0.004	0.020	0.037
$\mu _{T_{er}}$	0.76	0.03	0.71	0.81
$\sigma _{T_{er}}$	0.13	0.02	0.10	0.17

*Note*. The full reinforcement model had separate learning rates *η*^+^ and *η*^−^ for positive and negative prediction errors, two parameters to describe the non-linear mapping between the difference in values and the drift rate, a scaling parameter *v*_mod_, and an asymptote $v_{\max }$, one fixed threshold parameter *a*_fixed_, one value-modulation parameter *a*_mod_, and finally one non-decision time *T*_*e**r*_. Note that $\mu _{\eta ^{+}}$, $\mu _{\eta ^{-}}$, $\mu _{v_{\text {mod}}}$, and $\mu _{v_{\max }}$ were transformed for interpretability

**Table 8:** Group parameter estimates of the model of Pedersen et al. (2017)

Parameter	*M*	*SD*	2.5% percentile	97.5% percentile
$\phi (\mu _{\eta ^{+}})$	0.09	0.02	0.05	0.14
$\sigma _{\eta ^{+}}$	0.57	0.11	0.39	0.81
$\phi (\mu _{\eta ^{-}})$	0.17	0.03	0.12	0.25
$\sigma _{\eta ^{-}}$	0.47	0.13	0.24	0.75
$exp(\mu _{m})$	0.14	0.01	0.12	0.17
$\sigma _{m}$	0.41	0.08	0.27	0.60
$exp(\mu _{bp})$	0.013	0.005	0.005	0.023
$\sigma _{bp}$	1.321	0.237	0.894	1.821
$\mu _{bb}$	1.85	0.06	1.74	1.95
$\sigma _{bb}$	0.27	0.04	0.20	0.36
$\mu _{T_{er}}$	0.75	0.03	0.70	0.80
$\sigma _{T_{er}}$	0.13	0.02	0.10	0.17

*Note*. The model of Pedersen et al., (2017) had separate learning rates *η*^+^ and *η*^−^ for positive and negative prediction errors, a drift rate scaling parameter *m*, two parameters to define the trial-by-trial decrease of threshold, *bp* and *bb*, and one non-decision time *T*_*e**r*_. Note that $\mu _{\eta ^{+}}$, $\mu _{\eta ^{-}}$, *μ*_*m*_, and *μ*_*b**p*_ were transformed for interpretability

A graphical representation of the Bayesian hierarchical full reinforcement learning diffusion decision model (RLDDM) can be seen in Fig. 13. The model priors and hyper-priors were specified as:

$$\mu_{v_{\max}} \sim \mathcal{N}(0, 1); \sigma_{v_{\max}} \sim \mathcal{HN}(0, .5)$$

$$v_{\max} \sim \exp\left( \mathcal{N}(\mu_{v_{\max}}, \sigma_{v_{\max}}))\right.$$

$$\mu_{v_{\text{mod}}} \sim \mathcal{N}(-1, 1); \sigma_{v_{\text{mod}}} \sim \mathcal{HN}(0, .5)$$

$$ v_{\text{mod}} \sim exp(\mathcal{N}(\mu_{v_{\text{mod}}}, \sigma_{v_{\text{mod}}})) $$

$$ \mu_{a_{\text{fixed}}} \sim {\Gamma}(1, 2); \sigma_{a_{\text{fixed}}} \sim \mathcal{HN}(0, .5) $$

$$ a_{\text{fixed}} \sim \mathcal{N}(\mu_{a_{\text{fixed}}}, \sigma_{a_{\text{fixed}}}) $$

$$\mu_{a_{\text{mod}}} \sim \mathcal{N}(0, .1); \sigma_{a_{\text{mod}}} \sim \mathcal{HN}(0, .1)$$

$$a_{\text{mod}} \sim \mathcal{N}(\mu_{a_{\text{mod}}}, \sigma_{a_{\text{mod}}})$$

Priors and hyper-priors for *η*^±^ were the same as in the RL model, while for *T*_*e**r*_ they were the same as in the DDM.

## Appendix C: Results of parameter estimation

In this section, we report the results of estimation of the group parameters for the best-fitting models in each class of models (i.e., RL, DDM, RLDDM, and the models of Pedersen et al., 2017) (Table 5, 6, 7 and 8).

## Appendix D: Parameter recovery

Models in decision-making, and particularly RL models, can suffer from poor identifiability and parameter recovery, especially when the information content in the data is low (Spektor and Kellen, 2018). To alleviate this concern, we conducted a parameter recovery study for the most complex model, the one that is most likely to suffer from poor identifiability. We ran ten independent simulations based on the original dataset (i.e., with the same number of participants and trials per participant) and using the most likely samples of the estimated joint posterior distribution. We then estimated the group and individual parameters for each individual synthetic dataset as described in the Methods section.

We inspected the results in three different ways: (1) correlations across the samples of the group parameters (i.e., mean and standard deviations); (2) ability to correctly infer the group means; (3) ability to correctly infer the individual parameters, summarized as mean and median of the individual posterior distributions.

The correlations across samples can be seen in Fig. 14, obtained by averaging the correlation matrices across the independent simulations, together with the SD across correlations. The highest observed significant positive correlation (*ρ* = .19 , p < 0.05) was found between $\sigma _{v_{\max }}$ and $\sigma _{v_{\text {mod}}}$, while the highest significant negative correlation (*ρ*=−.15, p<0.05) was found between $\mu _{v_{\max }}$ and $\mu _{v_{\text {mod}}}$.

The posterior distributions of the mean group parameters for the ten independent simulations can be seen in Fig. 15. While most group means and standard deviations were successfully recovered, $\sigma _{v_{\max }}$ was sometimes underestimated.

Finally, recovery of individual-level parameters is shown in Fig. 16. Most parameters recovered well and showed a slight shrinkage to the mean (which is a feature of hierarchical models).
